# Reflections on the benefits and challenges of using co-produced artistic workshops to engage with young people in community settings

**DOI:** 10.1186/s40900-024-00575-1

**Published:** 2024-06-03

**Authors:** Laura Tinner

**Affiliations:** 1 Bristol Medical School, Population Health Sciences, Canygne Hall, Bristol, BS8 2BN UK; 2 Population Health Sciences. Bristol Medical School, University of Bristol, Bristol, BS8 2PN UK

**Keywords:** Inequalities, Adolescent health, Co-production, Creative methods, Qualitative

## Abstract

**Background:**

Despite increased focus on adolescence, young people’s voices are often undervalued and underrepresented in health inequalities research and policy. Through exploring young people’s priorities for their health and their community, we may begin to understand how public health interventions and policies can be more effective and equitable. Engaging with youth using art enables empowerment and self-expression on these complex topics.

**Methods:**

Creative workshops, co-produced with a young artist, were delivered at three youth centres to participants aged 11–18 years (*n* = 30) in disadvantaged areas of Bristol, UK. Participants engaged in art and were guided by a semi-structured topic guide through focus group discussion. Thematic analysis, supported by the young artist, was used to distil key policy priorities for young people to be delivered to the local authority.

**Results:**

The young people identified a list of key priorities. These were: (1) mental health, (2) feeling ‘forgotten’ as an age group and having safe city spaces to socialise, (3) the need for greater support for their education and career aspirations. I provide a brief summary of these priorities, but the focus of this article is on the critical reflections on this innovative way of engaging with young people about local policy. I provide key learning points for researchers looking to do creative public health work in community settings and involve marginalised young people.

**Conclusions:**

Art is a promising way of engaging with young people in community settings and elevating marginalised voices. Co-producing with a local young artist enriched the project and partially alleviated power imbalances. This approach has potential for involving different groups within local policymaking and priority setting around health inequalities.

**Supplementary Information:**

The online version contains supplementary material available at 10.1186/s40900-024-00575-1.

## Abstract (plain language summary)

This article talks about a project that was done in Bristol, UK. The project used art to help young people open up about local issues and about their health. The project involved workshops which took place in community centres and youth clubs, with a young artist leading the workshops. This article is less about what young people told us in those workshops and more about the process we went through, what the pros and cons of the study are and what other researchers can learn. The key lessons were: (1) art helps young people talk about complex and sensitive topics, (2) working with a local young artist helps young people open up and (3) it is important to work with people like youth workers and outreach workers when working in the community so we can make sure all young people have a chance at sharing their views and experiences.

**Supplementary Information:**

The online version contains supplementary material available at 10.1186/s40900-024-00575-1.

## Background

### Why engage with young people about health inequalities?

Adolescence refers to the period between childhood and adulthood, roughly between the ages of 12 and 19 [1]. In this article I refer to individuals of this age interchangeably as ‘youth’, ‘adolescents’ and ‘young people’, given the differential labelling of people of this age across academic research, local authorities, third sector and community organisations. Adolescence is a critical period in the life course for addressing health and social inequalities, as it is at this time that individuals can improve their life chances through education [2] and entry into the labour market [3]. Further, there has been building calls outlining reasons why adolescence should be a focus of public health investment, policy and research [4], particularly in relation to wider determinants of health and health inequalities [5–8]. There is also increasing recognition of the importance of youth participation and engagement in the research, both through policy and intervention development [9]. There are a number of useful guides about involving young people in research [10, 11]. A few notable qualitative studies on young people’s perceptions of health inequalities highlight how meaningful inclusion of young people can support a move toward pro-equity research and policymaking [6, 12, 13]. However, youth engagement in research too often commences after research questions, design and protocols are already defined, rather than involving young people from the beginning during priority setting [14].

Involving young people in policymaking has also gained unprecedented traction in the UK, developed from theoretical underpinnings of the Sociology of Childhood [15]. Common forms of involvement include youth consultations and formal initiatives such as youth parliaments or councils within local authorities or devolved governments [16, 17]. Further, by UK voluntary organisations such as *Children’s Rights Alliance for England*, *The Children’s Society* and *Investing in Children* have championed young people’s rights and involvement in decision-making processes. However, there are critiques related to tokenism [18] as well as whether such initiatives meaningfully involve young people from marginalised groups, including those from economically disadvantaged or ethnic minority backgrounds [16]. A further issue, not unique to young people’s involvement, is around the messy and complex world of knowledge mobilisation and evidence-based local policymaking [19–21]. This ‘evidence-policy gap’ has been debated widely in relation to public health, with a variety of examples of researchers frustrating experiences attempting to navigate local policy influence [19]. This literature provides important context for the challenges I encounter throughout this project.

Schools have a major role to play for young people’s health and involvement in research and policy [22]. Although, youth spend over 50% of their waking lives outside of school [23], so it should not be the only avenue through which to engage with young people. Further, conducting research outside of a school setting may have benefits in reaching marginalised young people who may not volunteer in school-based studies or be comfortable sharing their views among classmates and teachers. There is also evidence that of high levels of motivation and engagement in projects in alternative settings to schools, which some have suggested relates to youth feeling more ‘active, in control and competent’ in these settings ([24] p. 327, [25]). While this is not a new approach to gaining insights from young people [6] it is relatively underdeveloped in public health comparative to school-based research. This project set out to understand young people’s views of health inequality within the city that they live and deliver key priorities to local authority colleagues, therefore, engaging with youth physically *within* those community spaces was important.

### Why use art to engage with young people?

One method increasingly used within youth work and public involvement [26], although less so in public health research, is creative arts. There have been ongoing debates within research and policy surrounding the connection between arts and health [27–29], including an All Parliamentary Group Inquiry that proposed various strategies for embedding arts within health and social care [30]. These debates have stemmed from a recognition that engagement in cultural and creative activities has potential for promoting health and wellbeing. Critically, however, there remain challenges in evaluating these claims [27]. There is a lack of rigorous and systematic review work on the extent to which engagement in art can improve health [27], with the strongest evidence supporting the use of arts and music for social development in children and improved psychological factors in adults [31] and weak or a lack of evidence for the use of arts to reduce social inequalities and prevent physical and mental illness [27, 31].

Despite this evidence (or lack of) within health literature, there is still rationale for using art as a component to promote inclusive and meaningful and inclusive engagement in research, given its promising use in a number of co-production and participatory studies [28, 29, 31]. It is from this starting point within the literature that this study was borne – not as a way of using art to specifically improve young people’s health or as a project to test creative arts as a research method. Instead, I developed the project to explore young people’s experiences and perspectives about health and inequality within community settings and creative arts presented a promising tool (as suggested by my community organisation collaborators) to do that in a meaningful way.

Engaging with young people through art enables youth to express themselves on topics which might be abstract or complex [29]. It also shifts the gaze to the creative product, fostering less intense and invasive communication that is responsive to participants’ own meanings and associations [32]. Whilst there is no guarantee of full and active participation, creative methods have the potential for more collaborative data production [33], empowering young people to take part in research in a meaningful way, particularly if topics are sensitive. For example, young people may not want to verbalise their thoughts on certain topics in a direct way as is required by a traditional interview or focus group, but may still have a lot to say on topics such as mental health, inequality and poverty – which can be particularly challenging issues to discuss and all featured in this project. Through using art (even very simple colours and shapes) and metaphor [34], participants can then describe what they have created, why and what it means to them, which can then potentially uncover perspectives and experiences that may not have been from a direct question on that topic [34, 35]. This chimes with Langley et al.’s [29] special issue that synthesised cross-cutting themes of creative co-production studies, interpreting that creative methods enabled accessible and inclusive expression of complex ideas and fostered a relaxed and messy environment ideal for building relationships..Actively involving young people and people who support them in the design of the creative project also enhances the likelihood that sessions will be engaging, leading to benefit for both the participants and researchers. Access to creative arts opportunities is also an equity issue. Young people from disadvantaged backgrounds have limited opportunities to engage in art, particularly as UK austerity measures have led to reductions in art within school curriculums [36].

## Our approach

This project set out to form collaborations with the local authority and community youth centre partners and explore young people’s views on health inequalities to direct future research in this area. There was also interest from the local authority to hear young people’s priorities for local policy, given that most evidence used in this process was either quantitative and collected in schools. Discussions with local community groups also identified the need for fun and creative activities within the community, particularly given the fallout from the Covid-19 pandemic and considerable resource issues across the youth sector. Therefore, we collaboratively developed the following project objectives:

- To give young people the opportunity to take part in creative arts as a means of self-expression and storytelling
- To empower a young artist-facilitator in co-creating and leading research workshops
- To explore young people’s perceptions of health, inequality and their aspirations to inform future research
- To identify youth priorities for their health and living in the city that could inform local policy

## Methods

The study design was a workshop series that was co-produced with a young artist, comprising artistic exercises and focus group discussion. The aim of this article is to reflect on the process of: (a) engaging with marginalised youth in community settings, (b) using co-produced artistic workshops as an innovative method within public health research and (c) involving young people in local policy.

### Recruitment and sampling

Ten community youth groups across the city were contacted based on lists on the local authority website and social media. Most community group representatives responded but were unable to participate due to staff capacity or other priorities during the Covid-19 pandemic. The result was three workshops hosted at three different community groups within different areas of Bristol, representing different levels of deprivation.

The young artist (under 25 years) hired to co-produce the workshops was recruited through the Creative Youth Network [37] Alumni programme. A call was put out to the alumni, with a date by which to contact me expressing their interest and ideas for the workshop. Originally, I had conceived of the project as having several different artists run the sessions, but due to limited response, just one artist led all three sessions. The artist was paid for their time planning, co-producing and delivering the workshops, in keeping with local standards for freelance work. They were also given a reference for their CV. The artist signed a consent form that highlighted that they could withdraw from the project at any time, outlined confidentiality agreements in relation to the participants and clarified how the project would run. Although no formal training was given, our preliminary meeting involved an overview of public health research, young people and health inequalities, what the project was trying to achieve, ethical principles and research procedures the project should follow. We revisited this regularly if the artist had any questions.

The youth participants were recruited through the host community youth groups. Participants were eligible if they were aged between 11 and 18 years of age (broadly defined as the adolescent period and indicating secondary school age in the UK) and were able to provide parent/guardian consent to participate if they were under 16 years old. Young people were sent a participant information sheet for themselves and their parents, which was circulated by youth workers. In cases where English was not the first language of the parent/guardian, outreach workers engaged with families to explain the project aims, risks and benefits of taking part and obtained written consent. Parents either emailed the consent form or the young people brought the signed consent forms to the session.

Ensuring proper resourcing for the project and that the funds remained in the community was a key aspect of this work. Even where community groups were happy to be involved for free as they saw it as a worthwhile project, I discussed with them what type of funding would be most beneficial to them. All young people participating were given shopping vouchers as a thank you,, as well as a substantial pack of arts materials they each took home. Each community youth centre was remunerated to cover the costs of assisting with recruitment and for having a youth worker present at the session. The study also paid for room hire and catering costs of the community groups’ choice.

### The workshops

The workshops took place over the summer of 2021. The lead researcher had a series of meetings with the artist-facilitator to co-create a plan for the workshops. This included deciding on different artistic mediums; becoming familiar with our different perspectives and the research objectives; exploring the most engaging and appropriate activities for the young people; and designing a lesson plan for the workshop.

**Fig. 1:**
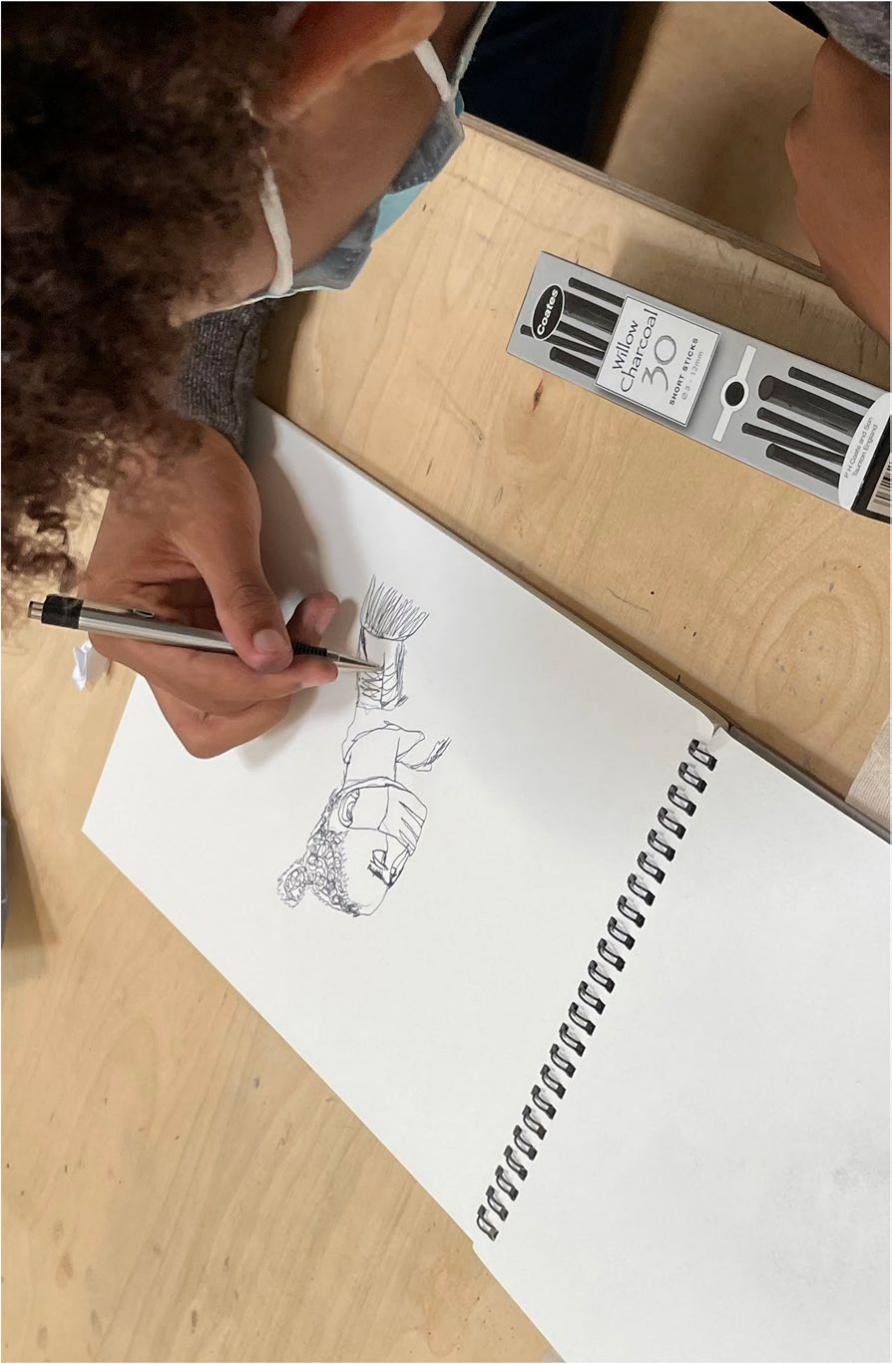
Workshop participant drawing what they think of when they think of health

**Fig. 2:**
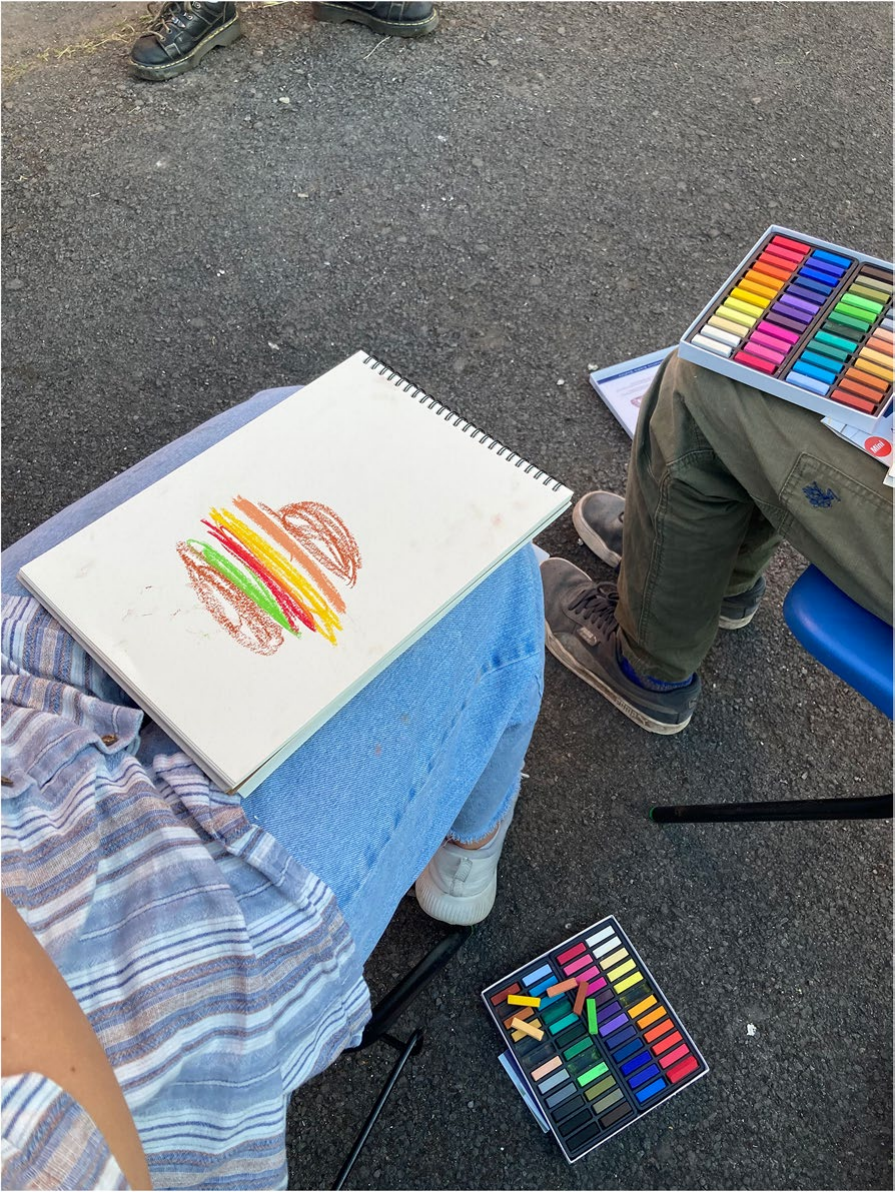
Workshop participant drawing what they think of when they think of 'food

**Fig. 3:**
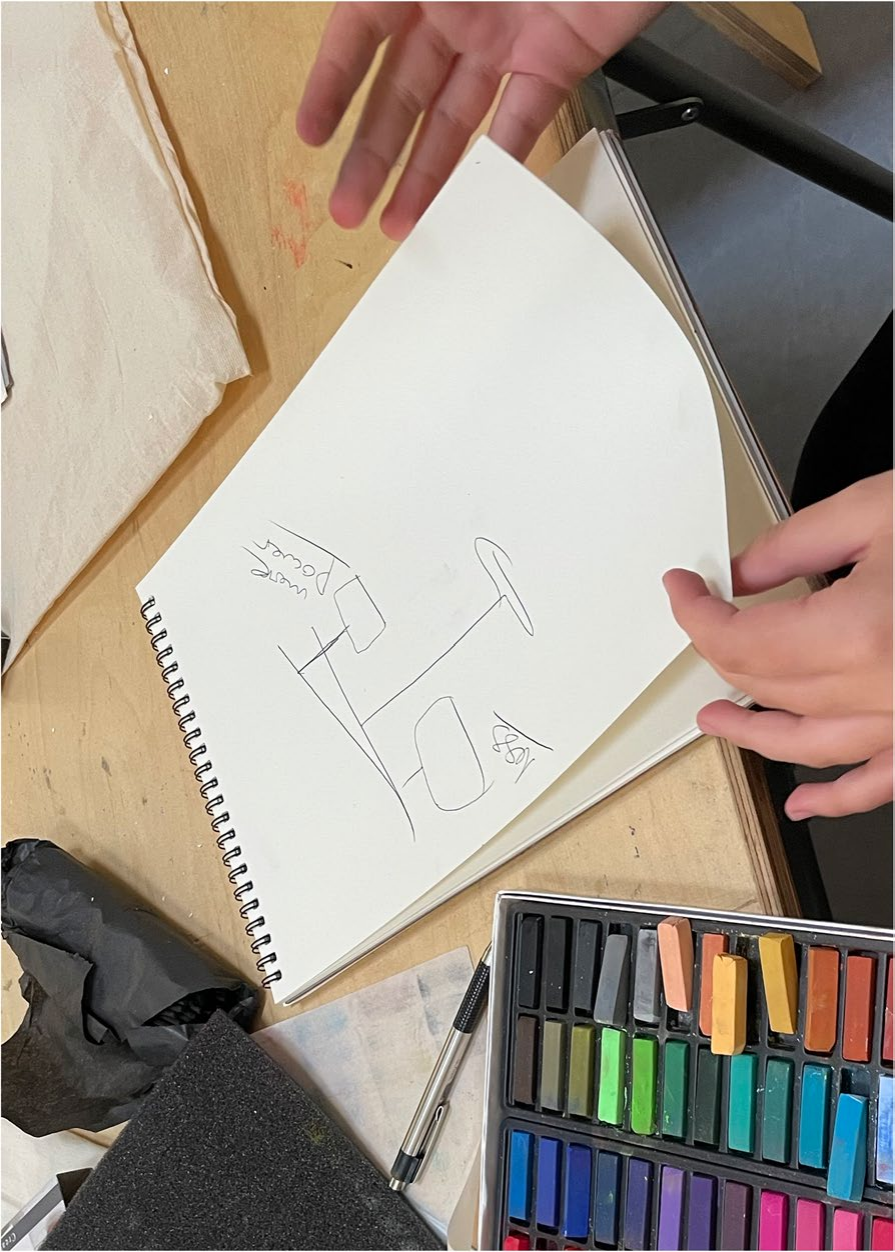
Workshop participant drawing what they think of when they think of inequality

The workshops were held in-person at the three youth centres. Upon arrival to each workshop, we handed out tote bags of artistic materials to participants which included charcoal, a drawing pen, a sketch book, coloured pastels, watercolour paint pallet and brush, which everyone was able to keep after the workshop. The session was split into two parts: the first being creative activities developed by the artist and the second being a focus group discussion. The artist led a variety of creative exercises that developed rapport and encouraged group engagement (drawing with eyes closed, drawing with the wrong hand, drawing the negative space), exposed participants to different techniques (continuous line drawing, shading, watercolour painting, charcoal use) and got participants to start thinking about the focus group topics. The final exercise before the focus group discussion was a word association game where participants chose a colour and drew for 20 s about words such as ‘exercise’, ‘drinking’, ‘eating’, ‘opportunity’, ‘inequality’ and ‘health’. Figures 1, 2 and 3 display some of the artworks young people created through these exercises.

After a short comfort break for snacks, the participants came back together for the focus group. The lead researcher started the recording, having confirmed with all participants that they were happy to be audio recorded. We used a semi-structured topic guide to frame discussions that was co-developed between the researcher and the artist. We used the word association game as a starting point for discussion, asking participants to expand on why they chose certain colours and shapes and what it expressed for them. The topic guide (Appendix 1) included questions about what young people thought about ‘health’, whether they thought of young people they knew as being healthy and why/why not. It went onto inequality topics, asking young people if they were aware of any differences in experiences of living in the city in relation to things like health, safety, behaviours, school, crime and Covid-19.

### Data analysis

The focus group discussions from the workshops were audio reordered using an encrypted Dictaphone. Recordings were transcribed by the lead researcher using the Braun and Clarke notation system [38]. We used reflexive thematic analysis [39] to inductively identify a core set of themes. The themes and concepts were discussed with the artist to clarify the interpretations from a non-researcher perspective.

The findings were presented at a local authority meeting of public health partners, who then took forward the recommendations into their practice work. The findings were also presented at the South West Public Health Scientific Conference in 2022 and at the UK Public Health Science Conference in 2022 [40]. Excerpts from the findings are presented in this paper where appropriate.

### Ethics

Ethical approval was obtained from the University of Bristol Health Sciences Faculty Research Ethics Committees (REF: 114,785).

As agreed prior to the workshop, the safeguarding procedures of the community group were upheld in the first instance as they were hosting the workshop on their premises and had ultimate responsibility for the young people. I had my own incident form to complete should there be an accident or a safeguarding incident as well as a distress protocol to follow should a young person become upset. In cases where something particularly sensitive was brought up (e.g., mental health, bullying), the young people were reminded that they could leave the session for a one-to-one chat with the youth worker if they wanted. I had a debrief with all the youth workers immediately after each workshop. They agreed to follow up with particular young people if they had brought up something sensitive or required follow up, and if necessary, their parent/guardian if they deemed appropriate. I also debriefed with my line manager the day after each workshop to clarify that no further involvement from the research team was required.

## Results

### Key policy and research priorities identified by young people

The young people across the three workshops identified a set of key priorities for their health and daily lives within the city, that they wanted the local authority to consider in their decision making. Firstly, mental health was highlighted as increasing problem among young people. Participants said that they were able to discuss mental health problems such as anxiety, depression and self-harm, but felt that the adults in the lives (parents, grandparents and teachers) were uninformed about mental health and therefore unable to support them. Secondly, young people across all the groups claimed that they fell into a ‘forgotten’ age group, with city spaces such as parks, city centre and the habour area being for either younger children or adults. Most workshop participants highlighted a priority for safe spaces to socialise over paid-for and structured activities. Finally, all youth felt uncertain about their future and many suggested that the local authority could encourage schools or other initiatives to support them in making education and employment decisions, particularly around age 16.

These priorities were used to direct my own research towards mental health inequalities, having previously focused more on health risk behaviours. The views of the young people were presented at a local authority public health meeting and feedback given to the young people around current and forthcoming policy strategies in the city. Now, I present the critical reflections and learning points of the approach.

### Benefits of the approach

#### Art as a communication and engagement tool

The artistic exercises proved to be an effective way of opening lines of communication with the young people. The participants spoke about potentially sensitive topics that the youth workers told us they had never mentioned previously, despite their efforts to talk to them about these issues. For instance, there were open discussions about mental health and the lack of support young people feel they have:

*Because before lockdown, a lot of people that I know were like, fine and now a lot of people that I know are into self-harming and things like that (Workshop 1, IMD Decile 5).*

I think adults need to be taught more about mental health and stuff like that. I think most of my teachers wouldn’t know about depression (Workshop 1, IMD Decile 5).

Our collaborators were surprised at how the young people discussed mental health and determined that the artistic techniques created the environment to allow them to talk about it. One community youth worker commented that this was particularly illuminating for the young men who usually attend the youth club to play football with their friends. Reflecting on what it was about the workshop that may have facilitated this, it could align with Langley et al.’s [29] findings that creative practice allow accessible self-expression that may mean that those not always comfortable with communicating verbally have avenues to get their perspective across. It may also have been the relaxed and informal atmosphere, with encouragement from the artist to have a go and fail that was a particularly enabling component for young men. Given the wide assumption that young men may struggle to talk about issues such as mental health more than young women [41], using creative arts to open lines of communication with young men could be explored in future work as it was beyond the scope of this project to probe or test this specifically.

The benefit of framing art as a method of communication as opposed to a skill that we were coming in to teach young people was that those young people who may not see themselves as particularly good at art could still be engaged. When I initiated the project, what I envisaged was that each young person would produce a piece of art that represented their understanding of health inequality. Through the co-production process, the young artist highlighted how that would likely not work in a one-off session for this age group. She also reminded me that the aim of the project was to get young people to talk about local issues and report their experiences for policy, so getting them too focused on finishing a piece would draw away from discussion. Instead, the artist suggested using short fun exercises using the artistic materials as a way of building confidence, socialising and learning a few tips related to art. This approach reduced the pressure on young people to be good at art or to translate their thinking about inequality into art. This experience strongly highlighted the power of art as a communication tool and the fact that to be used in public health research, there does not need to be a ‘finished’ product – and in fact the pressure of that objective can put young people off. This highlights a potentially important component that may be of use to remember for researchers hoping to engage in this work – the importance of giving participants permission to fail from the start [29]. I believe this created the optimum conditions for discussing sensitive or complex topics, as when young people became more relaxed and less concerned with getting it right, they appeared to be more active in the discussion [29]. It may have also contributed to reducing fears around getting things wrong in what they contribute in discussions around health and inequality. Critically, it may have been other conditions or mechanisms that fostered this rich engagement with inequality topics – this is something that should be explored in comprehensive evaluations of creative methods.

#### Mutual benefit, comfort and mitigating power imbalances

Collaborating with community groups and providing a meal and activity for local youth allowed the project to give something back to the community. Public health and inequalities projects should be initiated by asking multiple stakeholders what will best allow different people to participate in the research. I made concerted efforts to do this through several meetings with the community leaders from the start, to understand how the workshops would be best delivered for them, what they and their young people want to get out of it and what type of food would be appropriate. Establishing mutual benefit and understanding where different groups saw value in the project was hugely important. For example, one group were enthusiastic about young people’s views being heard at the local authority, another were interested in the artistic side of things and another were purely interested in having another way to reach marginalised young people they had been trying to get to come along to the community group. The flexibility and collaborative nature of this type of project meant that I could mould the workshop to what each group was hoping to achieve from the work.

The fact that the artist was not from the university and had attended group sessions at youth centres throughout their adolescence, I believe, enhanced their ability to build trust and create a relaxed atmosphere with the young people. Whereas I prioritised ensuring the consent forms were completed and guiding everyone through what to expect from the workshop, finding out young people’s priorities and delivery to the local authority, the artist was more focused on fun, getting to know the young people and connecting the young people with different types of art. My reflection is that had I independently delivered the workshops, it may have been a more formal atmosphere akin to a standard research setting, but as the artist had a differing perspective, we were able to deliver something appropriate to the setting and participants, that also upheld strong ethical research standards and uncover insightful and useful findings. This approach was also beneficial to the young artist’s skills development and confidence-building through gaining experience in leading a creative workshop. The community groups also valued the artist’s workshop delivery style as two of them hired them to conduct further work with their young people as a result of this project.

The combination of the community setting and the involvement of a young artist in planning and delivering the sessions resulted in high levels of engagement within the project. The young people were comfortable in the setting, which meant they were able to discuss critical issues to them. The young artist created a more informal and relaxed environment than I would have as an independent researcher. It also meant that she thought of some interesting ways of asking the young people things that I would not have, highlighting the benefit of not just her artistic skills but a non-researcher perspective who was closer to the participants’ age.

#### Elevating marginalised voices

Finally, delivering the project in community settings allowed marginalised voices to be heard at local policy level. Many public health researchers have strong links with schools, but there tends to be less formal and solid collaborations with community youth settings. If we aim to reduce inequalities and undertake impactful public health research that reaches those who need it most, it should be a priority to engage with people where they live and where the social determinants of health operate. This is not to say that school settings are not essential to public health research with young people. Rather, community youth centres are a valuable complement to this kind of research that has the opportunity to develop creative ways of engaging with the public and collecting data.

This benefit of engagement in community settings not only refers to marginalised groups of young people, but also marginalised opinions and thoughts of all young people that may not be easily discussed within a school setting. For instance, discussing topics such as health risk behaviours or critical comments about the school may be uncomfortable for young people in a school setting around peers and teachers:

*Yeah, most people have smoked at my school (Workshop 3, IMD Decile 2)*

*I think, maybe the whole year has tried alcohol (Workshop 1, IMD Decile 5)*

*Yeah I just finished school, but uni’s just, I dunno, when people ask me where I’m going I’m like, I don’t know- My school… Erm, [they haven’t] really supported me, to be honest (Workshop 2, IMD Decile 1).*

There were also other quotes from the workshops which challenged what local authority colleagues had determined from their work in school settings. For example, how young people experienced physical space around the city and felt ‘*forgotten*’ as an age group as a result. Further, young people prioritizing safe areas to sit and socialize over structured activities was not something that had been gleaned from school-based studies across the city:

I think this age, this sort of generation, it’s definitely the most left out, like people sort of forget about us (Workshop 1, IMD Decile 5).

There’s mostly just pubs. Like for older people and stuff, there’s not really much for young people (Workshop 2, IMD Decile 1).

We just want a little area, a safe like bench area or something to sit down and chill with your friends (Workshop 3, IMD Decile 2).

The outcome of this realisation was that the local authority would pursue qualitative work within community settings more regularly as much of the local strategy work had been informed by quantitative work and engagement in schools. This project identified that there were likely gaps in their understanding around how adolescents ‘belong’ in Bristol. Therefore, it was a major learning point for policy, practice and research that in order to meaningfully involve young people, we should look to multiple diverse settings outside of school. Feeding this information back to the community groups was a benefit, with youth workers claiming that the young people felt valued in the process by hearing how what they said was used by the local authority. Although feeding back to participants in this way is recommended and should be commonplace as good ethical practice [42], this positive response further highlighted to me that it should remain a priority to maintain the feedback loop. To advance this further, in future projects I will seek to understand what feedback would be valued to participants much earlier in the process.

Elevating marginalised voices is a key pillar to enhancing equity within research and policy. This project appeared to encourage inclusivity and access to research among some particularly marginalised young people and they all told me they had not engaged in research previously. However, I am unable to ascertain from this small project whether the draw for the young people was the ability to engage in research or local policy, the artistic activities, the community setting or it was the work of the youth workers to encourage the young people to engage. Therefore, although upon reflecting on the project it appears there is potential for this type of method to be used to enhance inclusivity and address inequalities in research, there is still limited evidence as to whether these approaches actually ‘work’ in pursuit of that goal [27].

### Challenges of the approach

#### Differing priorities

By far the greatest challenge of this kind of collaborative engagement work was that everyone had differing, and sometimes competing or evolving, priorities. For instance, I had my own aims to deliver the engagement project to as high a standard as possible, to collect data about young people’s experiences of inequality in Bristol and to engage with the local authority. The local authority was keen to hear the views of young people to inform the local policy, however, increasing time and resource restrictions and during the project and understandably focus on the Covid-19 pandemic made continued engagement difficult. This being a small, qualitative project (and the local authority had already had some engagement within schools) meant that it was a challenge to deliver that local policy impact I had hoped for. The lesson I learned from that was to establish stronger understanding through better communication with local authority partners at the beginning of the project. On reflection, I was not clear or direct on what the project could do for them, what strategy it would align with and exactly what I needed from them. Delays to the project due to local lockdowns also made it difficult to re-engage with the local authority after many months of the project being paused, which has taught me the value of regular communication even in the absence of project progress.

#### Informal practices

One of the benefits of engaging with young people in community settings is that they are placed right at the heart of the challenges and impacts of wider inequality, offering the potential for a deeper and more nuanced understanding of how health inequalities are experienced [43]. However, these settings are less well-versed in research than schools, and also the age ranges of young people attending is much more fluid. If the study took place in a school, we would have likely run workshops within year groups. Instead, we were led by the community groups and the young people they usually engage with, meaning a larger spread of ages and therefore differing levels of understanding about the concepts we were discussing. We navigated this through strong partnership working with the community youth workers and being ready to adapt the discussion. For example, the young artist had a longer list of words that she selected from for the word association game, dependent on the age of the young people.

Further, the governance process was less well-trodden ground for the community groups. A few participants turned up to the community group for the first time, attending with a friend, but did not have a completed consent form. These interactions are crucial for youth workers engaging in the community and it would go against their ethos to turn young people away. At times it was a challenge for them to understand the need for informed consent, for example, if the young person had registered to be part of the youth club and the youth workers had spoken to the parents previously. In a few instances, we had to work with the community groups to engage with the families ‘on the fly’ to ensure informed consent was obtained. The lesson I learned from that was to have contingency plans in place (e.g., extra information sheets and consent forms) and to communicate with the community groups throughout to ensure that ethical standards are upheld while allowing for the flexibility they need to make sure they do not miss opportunities to connect with young people.

#### Engaging with community groups and marginalised young people

Despite several community groups being interested in the project, the capacity and funding issues across the sector made it difficult for them to engage. There is need to add as much value as possible to every project and to ask collaborators what would make it appealing to them. For many it was funding for additional staff to support the group, for others it was for me to attend another session to discuss research with the young people. Undoubtedly, it will often be the most disadvantaged community groups, largely run by volunteers, that are unable to engage with research or public engagement projects. From an equity perspective, it is these groups we may want to speak with the most and could also receive the greatest benefit from creative arts and engaging with young people in the local area. Although standard practice to renumerate organisations for their time, I found that I did not have a detailed understanding of what it would take for these community groups to be involved, having previously worked with schools which often have set terms or volunteer to be involved. Allowing additional funds that may enable these groups to get involved – and getting an understanding of what those might be from the start—as well as being flexible to what organisations might need, is a huge lesson for any researcher doing public health work in community settings for the first time. Additional thought and care is needed here particularly within the current climate of community and voluntary youth organisations (especially smaller organisations) being under immense pressure financially [44].

We did not collect the individual-level participant characteristics on socioeconomic background, but instead were given an overview of representation from the community groups. The first workshop too place in the least disadvantaged area, comparatively, in this project (Workshop 1: *n* = 15, Decile 5) using the Index of Multiple Deprivation [45]. Workshop 2 (*n* = 8 Decile 2) and Workshop 3 (*n* = 7, Decile 1) were based in comparatively more disadvantaged areas. Across the groups, there was some level of diversity based upon race/ethnicity (*n* = 20 White British and *n* = 10 Black British), age (range between 11–18 years) and gender (*n* = 14 female, *n* = 14 male, *n* = 2 gender non-binary). Therefore, through our collaboration with local stakeholders, we were able to engage with young people who could be considered ‘marginalised’ based on their socioeconomic background and race/ethnicity. Many of the participants were also marginalised from the youth work sector. Namely, this applied to one workshop that was designed for young women and girls in the most disadvantaged community, the majority of whom were from ethnic minority backgrounds. The youth workers had said that most activities for their age group had been ‘open access’ events, which often were well attended by boys who came along to football. The community group had struggled to engage with young women and girls due to the nature of the activities but also because many families were not comfortable with their daughters attending mixed-gender events.

It was only possible to engage with these marginalised groups in this project because of a few key outreach workers who spent time speaking with families to: (a) ensure parents could give informed consent, particularly where English was not the parents’ first language, (b) ensure the young people and families felt comfortable it would be suitable and the youth worker would be present, (c) accompany the young people to the workshop from their homes. While I could have reverted to ‘easier’ recruitment channels and or worked with a more established youth club to deliver the project quicker, that would have likely resulted in lower diversity and inclusion. It was a lot of extra work for the community group that had not been accounted for in the initial proposal, but was crucial for reaching those young people – all of whom told me they had not been involved in research before or had had their views heard about where they lived (at school or otherwise). For future work, I would ensure additional funds and time to support the engagement from outreach workers. They saw the workshops as valuable as it gave them a reason to engage with families and young people they had been struggling to connect with, but there is no denying it cost them additional time and resource to include those marginalised young people that should be accounted for.

## Discussion

This project has contributed to a building evidence base around the use of art for involving marginalised people in research and policy [46, 47]. There was overwhelming feedback from the young people and the youth workers that using art meant that the participants felt at ease and were able to open up about their understanding of health and inequality. These types of engagement methods are used widely in the youth work sector to empower young people and establish relationships. It is less used within research but has huge potential when children and young people are the intended population. The policy impact of this work was limited. It is clear that there are additional considerations for involving young people in the policy cycle, including better knowledge exchange and communication as well as weaving in the involvement work with current policy priorities [16]. The initiatives to include young people in research and policy are promising, as well as efforts to ensure those on the margins are fully represented [11, 16]. This article provides insight into the challenges of such an approach.

This project also highlights the power of co-producing the artistic engagement work. The young artist not only contributed skills and knowledge about art that were needed for the workshop, but they acted as peer researcher with ‘insider knowledge’ to develop a rapport with the young people [48]. Peer researchers are increasingly used to reduce the power imbalance between adult researchers and young people and can help elicit more meaningful engagement from participants [48]. The artist connected with the young people in ways that would not be possible for me as a researcher. Having the perspective of an artist, not just as a young person, was also incredibly enriching in terms of framing questions and engaging with the young people in ways I had not thought of. This experience has inspired me to continue to explore interdisciplinary working to strive for high quality engagement and research on health inequalities. Creative interdisciplinary models are beginning to be explored with the aim of tackling complex problems such as health inequalities, for instance, trans-disciplinary placements [49] and ‘sandpit’ style workshops [50]. This small engagement project highlights the arts as an underutilised discipline that could promote equity and through collaborating on health research, which is worth embracing.

Finally, this project provides key learning points for researchers looking to do public health engagement work or research in community settings and include diverse groups of people. It is increasingly being appreciated that to involve people from marginalised groups, researchers need to think creatively and be flexible to community needs [46]. It involves working collaboratively with stakeholders within the community and often takes a lot longer to recruit fewer participants than would be reached in schools. Those efforts are essential because if we are going to make in-roads into reducing health inequalities, those ‘hardly reached’ groups [51] must be included and feel empowered to talk about their lives.

## Conclusions

Art is a promising method for engaging with young people about difficult topics, but it takes extra effort and creativity to ensure the most marginalised young people are in the room and contributing. Co-producing the workshops with a young artist was essential for alleviating power imbalances and framing questions in more engaging ways. Being adaptable to community needs, maintaining that focus on engaging with those young people that are ‘hardly reached’ and not reverting back to ‘easier’ recruitment channels are key learning points from this project that I see as hugely valuable for public health equity work.

### Supplementary Information

**Supplementary Material 1.** 

## Data Availability

The qualitative data is restricted and available from the corresponding author at reasonable request and formal process.

## Abbreviations

UK: United Kingdom
CV: Curriculum Vitae
USA: United States of America
IMD: Index of Multiple Deprivation
PE: Physical Education
GCSE: General Certificate of Secondary Education
