# Supplementary material for: Reflections on the benefits and challenges of using co-produced artistic workshops to engage with young people in community settings
Source: Res Involv Engagem. 2024 Jun 3;10:51. doi: 10.1186/s40900-024-00575-1 (PMC11149207; doi:10.1186/s40900-024-00575-1)
Supplement: Supplementary file 1 — Supplementary Material 1. [file 40900_2024_575_MOESM1_ESM.docx]

**Appendix 1: Discussion questions for workshops**

Health

- What do you think of when you think of health?
- Do you think young people are healthy in Bristol?
- Do you think you are healthy?
- Do you think it’s easy or difficult for young people to be healthy in Bristol?
- What do you think are the main health issues for people your age?
- What kind of healthy/unhealthy behaviours do you think people your age engage in?

Inequality

- What is it like to live in Bristol [or area of Bristol]?
- What do you think of when you think of opportunity?
- Do all young people in Bristol have the same opportunities?
- What do you think about inequality?
- Has the pandemic impacted any of this?
- What would you like the council to know about your lives if you could tell them anything?
- What would you like to see for young people? Is there anything that would help you?
